# Neurobehavioral Associations with NREM and REM Sleep Architecture in Children with Autism Spectrum Disorder

**DOI:** 10.3390/children9091322

**Published:** 2022-08-30

**Authors:** Jennifer Nguyen, Bo Zhang, Ellen Hanson, Dimitrios Mylonas, Kiran Maski

**Affiliations:** 1 Department of Neurology, Division of Child Neurology, University of Rochester Medical Center, Rochester, NY 14642, USA; 2 Department of Pediatrics, Division of Developmental and Behavioral Pediatrics, University of Rochester Medical Center, Rochester, NY 14642, USA; 3 Department of Neurology, Boston Children’s Hospital, Harvard Medical School, Boston, MA 02115, USA; 4 Biostatistics and Research Design Center, Institutional Centers for Clinical and Translational Research, Boston Children’s Hospital, Harvard Medical School, Boston, MA 02115, USA; 5 Department of Medicine, Boston Children’s Hospital, Boston, MA 02115, USA; 6 Department of Psychiatry, Massachusetts General Hospital, Harvard Medical School, Boston, MA 02115, USA; 7 Athinoula A. Martinos Center for Biomedical Imaging, Charlestown, MA 02129, USA

**Keywords:** autism, sleep, behavior, NonRapid Eye Movement sleep (NREM), Rapid Eye Movement (REM), slow wave sleep, children

## Abstract

Objective: Insomnia and daytime behavioral problems are common issues in pediatric autism spectrum disorder (ASD), yet specific underlying relationships with NonRapid Eye Movement sleep (NREM) and Rapid Eye Movement (REM) sleep architecture are understudied. We hypothesize that REM sleep alterations (REM%, REM EEG power) are associated with more internalizing behaviors and NREM sleep deficits (N3%; slow wave activity (SWA) 0.5–3 Hz EEG power) are associated with increased externalizing behaviors in children with ASD vs. typical developing controls (TD). Methods: In an age- and gender-matched pediatric cohort of *n* = 23 ASD and *n* = 20 TD participants, we collected macro/micro sleep architecture with overnight home polysomnogram and daytime behavior scores with Child Behavior Checklist (CBCL) scores. Results: Controlling for non-verbal IQ and medication use, ASD and TD children have similar REM and NREM sleep architecture. Only ASD children show positive relationships between REM%, REM theta power and REM beta power with internalizing scores. Only TD participants showed an inverse relationship between NREM SWA and externalizing scores. Conclusion: REM sleep measures reflect concerning internalizing behaviours in ASD and could serve as a biomarker for mood disorders in this population. While improving deep sleep may help externalizing behaviours in TD, we do not find evidence of this relationship in ASD.

## 1. Introduction

Sleep difficulties are very common in children with autism spectrum disorders (ASD) with a prevalence of 40–93% [1,2]. At the same time, children with ASD also have significantly more daytime behavioral issues than their typically developing peers (TD), further increasing disease burden [3,4]. Numerous studies have shown associations between parent report and objective measures of decreased sleep quantity and quality in children with ASD and higher levels of behavioral issues [5,6,7]. Studies of alterations in NonRapid Eye Movement sleep (NREM) and Rapid Eye Movement (REM) sleep in ASD compared with TD vary in the literature. Using gold-standard polysomnogram (PSG) testing, Buckley et al. reported that young children with ASD (mean age 4.8 years) have reductions in REM sleep and increases in NREM slow wave sleep stage [8] compared with TD. However, Tessier et al. found no REM differences between ASD and TD groups in older pediatric cohorts [9], and Lehoux et al. showed only ASD topographic differences in NREM slow-wave activity (SWA; frequencies range 0.5–4 Hz) and TD [10]. Differences in participant age, cognitive status, medication use, and/or sleep analysis techniques may contribute to inconsistent findings. 

Sleep is critical for mood regulation and behavioral functioning in adults and children [11,12,13,14]. In particular, there is converging evidence from human and animal studies that normal REM sleep is critical for emotional memory consolidation and emotional reactivity [15,16]. A study of healthy adults showed that increases in REM prefrontal theta power activity are associated with improved emotional memory consolidation [17] and that abnormal increases in faster REM frequencies (frontal REM gamma activity) reflect amygdala reactivity on fMRI studies [18]. A number of pediatric studies also report associations between NREM 3 (N3, deep sleep) and NREM SWA and externalizing behaviors in TD children and those with pediatric sleep disorder studies [19,20,21]. Insight into associations between REM and NREM sleep with problematic behaviors in children with ASD could identify potential objective biomarkers of mood or disruptive behavior disorders in a population with known restricted communication skills as well as serve as potential targets for sleep-based therapeutics. 

In this study, we use home PSG to collect objective sleep architecture and quantitative EEG power in N3 and REM sleep to study associations with problematic behaviors reported on the parent-completed Child Behavior Checklist [22] among TD and ASD participants 9–16 years old. We hypothesize that in ASD participants (1) REM sleep alterations (less REM sleep percentage, less theta power and higher beta power vs. TD) will be associated with an increase in internalizing behavior scores and (2) reductions in N3 percentage (deep sleep relative to total sleep time) and NREM SWA (0.5–3 Hz spectral power; a marker of deep sleep) vs. TD will be associated with an increase in externalizing behavior scores. 

## 2. Methods

Data for this study result from the secondary analyses from a prior study on the effects of sleep and memory consolidation in pediatric ASD, and full methods are detailed in this prior publication [23]. We here describe only measures relevant to the current study. We conducted analysis on data from enrolled participants ASD and TD participants who completed a one-night home polysomnogram (PSG) and nonverbal IQ (NVIQ) testing and whose parent/guardian completed the CBCL survey. 

### 2.1. Participants

ASD participants ages 9–16 years met diagnostic ASD cutoff scores on the Autism Diagnostic Observation Schedule (ADOS) [24] and the Autism Diagnostic Interview, Revised (ADI-R) [25]. In addition, we required participants with ASD to have a nonverbal intelligence quotient (NVIQ) < 1.33 standard deviations below the mean (a standard score of 80 or higher) on the Differential Abilities Scales II (DAS-II) [26]. We recruited age-matched TD control participants via community newspaper ads and a classified advertisement website (Craigslist). 

The exclusion criteria for all participants are detailed in our prior work [23]. 

### 2.2. Procedure/Measures

Participants and parents/guardians completed study consent/assent and cognitive and neuropsychological testing in the Clinical & Translational Study Unit (CTSU) at Boston Children’s Hospital. Approximately 1–3 weeks later, the participants had a home PSG. Participants were instructed to avoid daytime naps during the period of study participation. 

### 2.3. Home PSG

Home PSG recordings were collected using an Embla A-10 ambulatory PSG system (Medcare Systems, Buffalo, NY, USA) with a standard montage including seven channels of EEG (F1, F2, C3, Cz, C4, O1, and O2), two of electro-oculography (EOG) and two of electromyography (EMG). All channels were digitally recorded at 200 Hz. 

### 2.4. Cognitive and Behavior Measures

We obtained nonverbal IQ scores (NVIQ scores) from participants using the Differential Abilities Scale II (DAS-II) [26], a battery of cognitive and achievement tests validated for school-age children 6 to 17 years. The score is calculated as a standard score with mean of 100 and standard deviation of 15. This test has been used to demonstrate cognitive abilities in children with autism [27].

The Child Behavior Checklist (CBCL) [22] is a well-established and widely used parent-completed measure of emotional, behavioral and social problems in children and adolescents aged 6–18 years. It comprises several subscales (Withdrawn Somatic Complaints, Anxious/Depressed, Rule-Breaking Behavior, Social Problems, Thought Problems, Attention Problems, and Aggressive Behavior) and includes three summary scale scores (Internalizing, Externalizing, and Total Problems). The CBCL Internalizing Score (CBCLi) reflects mood disturbance (including anxiety and depression) and social withdrawal. The Externalizing Score (CBCLe) reflects rule breaking and aggressive behaviors. 

The Children’s Sleep Habits Questionnaire (CSHQ) is a 35-item questionnaire validated in children ages 4–10 y [28] and previously used to assess sleep problems in ASD ages 2–18 years [29]. The CSHQ includes domains of bedtime resistance, sleep anxiety, sleep onset delay, night awakenings, daytime sleepiness, and parasomnias. Scores greater than 40 reflect clinically significant sleep problems. 

## 3. Data Analysis

### 3.1. Sleep Analyses

A physician board certified in sleep medicine (KM) scored sleep recordings in 30-s epochs using standard criteria [30], blinded to participants’ group and behavior scores. Sleep onset latency (SOL, time to fall asleep), wake time after sleep onset, total sleep time, sleep efficiency (time asleep/time in bed), and percentage of time (relative to total sleep time) spent in sleep stages NREM stage 1 (N1), NREM stage 2 (N2), NREM stage 3 (N3), and REM were calculated. Sleep recordings were then preprocessed and further analyzed using a BrainVision Analyzer 2.0 (Brain Products, Munich, Germany) and MatLab R2010a (The Math Works, Natick, MA, USA) software. We referenced EEG data to linked mastoids and filtered the EEG at 0.3 to 35 Hz. We manually rejected artifacts by visual inspection. More NREM epochs were rejected due to noise in the ASD group, but this did not significantly differ by group (TD: 8.8 +/− 8.3, ASD: 17.2 +/− 18.4. Wilcoxon rank–sum test z = 0.90, *p* = 0.34). Spectral power density was calculated by fast Fourier transform, applying a Hanning window to successive 3 sec epochs of NREM sleep with 50% overlap. We calculated spectral power for slow wave activity (SWA, 0.5–3 Hz), theta (4–7 Hz), alpha (8–11 Hz), sigma (12–15 Hz), and beta (16–20 Hz) frequency ranges. We did not evaluate gamma range activity (typically defined as >30 Hz) due to EEG low pass filters set at 35 Hz. For reporting, we present mean NREM SWA collected from all electrodes and REM theta, alpha, and beta activity from frontal leads (F1, F2). We chose to focus analysis on frontal leads in REM based on prior work suggesting important REM sleep emotional processes emerging from this region [17,31]. Unfortunately, since this analysis was conducted, the raw EEG data are no longer accessible to us due to corruption issues. We are unable to provide metrics of artifact rejection in REM sleep or power in individual electrodes. 

### 3.2. Statistical Analyses

We maintained the data in a REDCap database and performed data analyses using SPSS for Windows (version 19; IBM Corp, Armonk, NY, USA). We visually inspected data to check for normality and potential outliers (points that extend more than 1.5 box-lengths from edge of box plot). We report participants’ demographic, sleep, and cognitive and behavioral test results as means and standard deviations, and unpaired two-tailed *t*-tests were applied for group comparisons. For non-normal data, we report medians with minimum and maximum values and used the Wilcoxon rank-sum test for group comparisons. For categorical and ordinal data such as gender and medication use, we used Fisher’s exact test for comparisons. We performed group comparisons and within-group associations using linear regression models with and without NVIQ and medication use (scored as yes/no). We included these confounding variables in the model based on literature showing their influence on sleep architecture and behavior outcome measures [8,32,33]. In this way, we evaluated the hypothesized relationships between specific NREM sleep and REM sleep measures of interest in the ASD group and TD group separately: REM and N3 percentages of total sleep time, global NREM SWA, frontal REM theta activity, frontal REM beta activity, and the dependent variables of interest (CBCL externalizing and internalizing behavior scores). To control the false discovery rate in multiple comparisons, we implemented the Benjamini–Hochberg procedure with a false discovery rate of 0.10. 

### 3.3. IRB

This study was approved by the Boston Children’s Hospital Institutional Review Board. All participants provided assent, and their parents gave written informed consent to be included in this study prior to the collection of data. The data are not publicly available as informed consent/assent was not obtained from participants for data sharing.

## 4. Results

### 4.1. Participant Characteristics

All 23 enrolled participants with ASD and 20 of the 23 enrolled TD participants completed the sleep testing, NVIQ testing, and questionnaire data. We here report demographic, cognitive, and questionnaire data in Table 1. There were no significant differences in mean age or reported gender between groups. Participants with ASD had lower standard NVIQ scores [mean 103.3] than TD participants [112.7] *p* = 0.05, but all scores were within normal range. No participants in the TD group reported medication use, but 39% of ASD participants reported taking a medication at time of study. Reported medications included melatonin (*n* = 1), SSRI or antipsychotic (*n* = 3), stimulants (*n* = 4), and clonidine (*n* = 1). Participants with ASD had significantly higher scores on the CBCLi, CBCLe, CBCL total problems, and CSHQ than TD participants (*p* ≤ 0.001). 

### 4.2. Sleep Architecture

We report sleep architecture group comparisons in Table 2. PSG lights off and lights on were similar between groups (ASD: average 21:04 lights off and 07:34 lights on; TD: average 20:52 lights off and 07:15 lights on). ASD participants had more problematic sleep with longer sleep onset latencies, longer wake time after sleep onset, and poorer sleep efficiency even after adjusting for medication use and standard NVIQ scores (*p* < 0.05). Otherwise, NREM and REM sleep architecture assessments were comparable between the two groups.

### 4.3. Relationships between Daytime Behaviours and REM and NREM Sleep Stages

**Figure 1:**
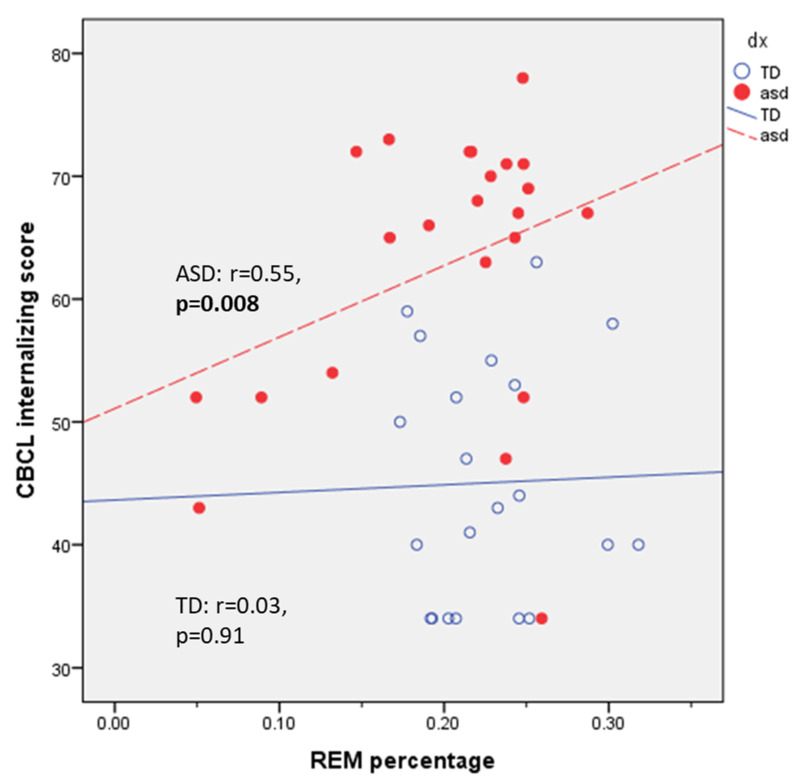
REM sleep percentage correlates with Child Behavior Checklist internalizing (CBCLi) score in ASD group but not TD. Bolded *p*-values represent significance *p* < 0.05.

On univariate testing, ASD participants demonstrated a significant positive correlation between CBCLi and mean REM percentage (r = 0.55, *p* = 0.008, Figure 1). There were modest but non-significant positive correlations between CBCLi scores and REM frontal theta power (r = 0.59, *p* = 0.06) and REM frontal beta power (r = 0.46, *p* = 0.16). On univariate testing, we found an inverse correlation between CBCL externalizing scores and global NREM SWA power that met trend significance in the TD group (r = −0.4, *p* = 0.08) and no correlation in the ASD group (r = −0.08, *p* = 0.72).

We report linear regression results in Table 3. Correlations between CBCLi and REM percentage, REM frontal theta power, and REM frontal beta power became significant when controlling for NVIQ and any medication use (p’s < 0.04). We found no significant associations between CBCLi and REM percentage, REM frontal theta power and REM frontal beta power in the TD participants. Adjusting for NVIQ and any medication use, the inverse association between NREM SWA power and CBCLe scores met significance in the TD group only (*p* = 0.01). We did not find any associations between CBCLe and N3 percentage in either group. All significant results remained significant after false discovery testing. 

## 5. Discussion

In this study, we find expected differences in sleep quality with longer sleep onset latency and decreased sleep efficiency in the ASD group vs. TD, but this sleep disruption did not alter the studied macro/micro NREM or REM sleep architecture as we predicted. However, the associations between REM and NREM sleep and daytime behaviours differed between the ASD and TD groups, albeit in unexpected ways. In the ASD group, CBCLi scores were associated with increased REM percentage of total sleep time, REM theta power, and REM beta power. We found no specific NREM sleep features associated with CBCLe in the ASD group; however, TD participants showed an inverse relationship between CBCLe and NREM SWA power. Taken together, increased REM sleep may reflect internalizing behaviours and/or mood disorder symptoms, whereas measures of deep NREM sleep are independent of problematic externalizing behaviours in ASD. Strengths of our work include well-matched samples for age and gender, the inclusion of known confounders (medication use and NVIQ), validated behavioural outcomes, well-phenotyped participants, and the use of home PSG for more naturalistic sleep environment than in-lab testing. 

In studies with school age, adolescent, and/or young adult ASD and TD participants [34,35,36], researchers also found no REM percentage differences between groups. Initial findings of REM sleep deficits in ASD prompted an open-label trial of donepezil to increase REM sleep [37]; however, our work suggests that more REM sleep may not be necessarily a good thing as REM features positively correlate with CBCLi. Increases in REM sleep density have been previously reported in adults with depression along with other REM alterations (frequency of rapid eye movements per REM period, shorter REM sleep onset latency) [38,39,40,41]. Plausibly, the association between REM sleep and internalizing behaviours in ASD could reflect underlying cholinergic neurochemical changes that may produce depression [42,43] and increase REM sleep [44]. To more fully elucidate this relationship in ASD, future research should consider additional measures of REM sleep including REM sleep stability (arousal/wake count in REM sleep, REM bout duration, number of REM bouts), rapid eye movement density, and frontal gamma activity (a marker of central adrenergic activity previously shown to be associated with amygdala activity and emotional reactivity [18]) as predictors of depression and anxiety. If our results are replicated, findings of increased REM sleep in people with ASD could be a useful objective biomarker of co-morbid mood disorder. Alternatively, cognitive neuroscientists have highlighted the importance of REM sleep for emotional memory processing, emotional reactivity, and the formation of emotional memories [17,18,45]. Increases in REM sleep in the ASD group may be serving to “self-treat” those with brewing mood dysfunction assuming that the REM sleep is in fact “normal”. 

While we found no differences in NREM SWA across all leads averaged together between participants with ASD and TD, differences may have been more apparent if we had studied recording from specific leads. For example, young children with ASD (mean age 4.6 years) had reduced SWA activity in the occipital leads during N3 sleep vs. a clinically referred TD group [46]. Similarly, young adults with ASD (mean 22.8 years) showed less SWA activity during NREM sleep in parietal occipital leads vs. a TD group [47]. In terms of associations between SWA and behaviour, our NREM SWA findings are consistent with a recent abstract reporting negative associations fronto-occipital NREM SWA with externalizing behaviours in TD children ages 5–12 years [19]. This may reinforce literature showing benefits of deep sleep in TD children. In a meta-analysis of research published over a 15-year period, no differences in NREM SWA were present between children/adolescents with ADHD and TD controls [48]. Thus, an association between externalizing behavior and deep sleep in ASD may be unlikely as well. Plausibly, these relationships may be more apparent with the use of high-density EEG and topographic mapping of SWA as shown in recent ADHD research [49]. 

Our study has some additional limitations. First, the ASD participants were high functioning, and these results may not be generalizable to all children with ASD. Second, although we did control for medication use in the ASD participants in our data analysis, we could not exclude medication use or stop medications during our study. Thus, unmeasured medication effects could be contributing to our reported results. Third, our study is small, and our findings need validation in large samples of patients. Lastly, we did not use an adaption night, and first-night effects could have influenced the sleep findings.

## 6. Conclusions

Clinically, externalizing behaviours and sleep disturbances are common complaints among parents of children with ASD, but we did not find justifications to increase deep sleep. If replicated in larger cohorts, we believe our REM sleep and internalizing association findings could help clinicians and researchers identify objective biomarkers of behavioural mood co-morbidities in children with ASD, a group in whom accurate reporting of symptoms can be challenging due to communication and social deficits. Such biomarkers could be helpful in identifying patients who may benefit from therapeutic interventions and aid in identifying homogeneous cohorts for future clinical trials. 

## Figures and Tables

**Table 1:** Participant Characteristics.

	ASD (*n* = 23)	TD (*n* = 20)	*p*-Value
Age (mean)	11.6 (2.0)	12.7 (2.1)	0.10
Gender (%male)	91.3	90	1
Standard Nonverbal IQ	103.3 (11.9)	112.7 (17.6)	0.05
Medications (%yes) *	39	0	**0.004**
CSHQ total score	49.2 (8.8)	39.9 (4)	**<0.0005**
CBCL internalizing behaviors	62.7 (11.3)	45.0 (9.7)	**<0.0005**
CBCL externalizing behaviors	53.9 (10.1)	43.6 (9.2)	**0.001**
CBCL total score	62.9 (8.6)	41.1 (10.9)	**<0.001**

* Any medications refers to any medications or substances used by participants including stimulants, antidepressants, clonidine, or melatonin. Mean (±SD) or frequency in percentage presented. CSHQ = Children’s Sleep Habits Questionnaire; CBCL = Children’s Behavioral Checklist. Bolded *p*-values represent significance *p* < 0.05.

**Table 2:** Sleep Architecture Comparisons.

	ASD (M ± SD)	TD (M ± SD)	Unadjusted *p*-Value	Adjusted *p*-Value
Time in Bed (min)	593.1 (65.2)	505.4 (70.3)	**<0.0005**	**0.001**
Total Sleep Time (min)	508.3 (59.8)	471.7 (68)	0.07	0.30
Sleep Onset Latency (min)	34.1 (23.1)	12.8 (9.9)	**<0.0005**	**0.002**
Wake After Sleep Onset (min)	50.7 (39.6)	20.9 (13.1)	**0.002**	**0.003**
Sleep Efficiency (%)	86.2 (6.6)	93.3 (2.2)	**<0.0005**	**<0.0005**
Number of wakings	12.7 (10.9)	6.6 (5.1)	**0.02**	0.08
N1%	3.8 (2.5)	3.0 (1.7)	0.24	0.25
N2%	47.1 (10.2)	45.1 (7.7)	0.46	0.92
N3%	29.1 (8.2)	29.1 (6.2)	0.99	0.32
REM (%)	20.0 (6.6)	22.7 (4.2)	0.11	0.07
REM latency (min)	142.7 (69.7)	115.3 (39.6)	0.11	0.13
Frontal REM theta power (µV^2^/Hz^−1^)	18.6 (9)	19.1 (8.2)	0.90	0.86
REM beta (µV^2^/Hz^−1^)	3.3 (1.6)	3.9 (1.3)	0.34	0.23
Global NREM SWA power (µV^2^/Hz^−1^)	283.01 (164.5)	216.41 (124.0)	0.16	0.21

Adjusted *p*-value reflects inclusion of standard NVIQ and any medication variables in linear regression model. Bolded *p*-values represent significance *p* < 0.05.

**Table 3:** Associations between REM and NREM Sleep and CBCL Scores within Groups.

Predictors	Outcomes	Unadjusted Association Statistic	Unadjusted *p*-Value	Sig. with FDR	Adjusted Association Statistic	Adjusted *p*-Value	Sig. with FDR
ASD							
REM percentage	CBCLi	F = 7.59	**0.01**	**Yes**	F = 7.05	**0.02**	**Yes**
Frontal REM theta power (µV^2^/Hz^−1^)	CBCLi	F = 4.846	0.06	No	F = 7.53	**0.03**	**Yes**
Frontal beta power (µV^2^/Hz^−1^)	CBCLi	F = 2.35	0.16	No	F = 6.29	**0.04**	**Yes**
SWS percentage	CBCLe	F = 2.51	0.13	No	F = 2.28	0.15	No
NREM SWA power (µV^2^/Hz^−1^)	CBCLe	F = 3.43	0.08	No	F = 0.078	0.78	No
TD							
REM percentage	CBCLi	F = 0.002	0.97	No	F = 0.018	0.89	No
Frontal REM theta power (µV^2^/Hz^−1^)	CBCLi	F = 0.007	0.93	No	F = 0.659	0.44	No
Frontal beta power (µV^2^/Hz^−1^)	CBCLi	F = 0.158	0.67	No	F = 0.917	0.36	No
SWS percentage	CBCLe	F = 0.162	0.69	No	F = 0.107	0.75	No
NREM SWA power (µV^2^/Hz^−1^)	CBCLe	F = 0.128	0.72	No	F = 7.78	**0.01**	**Yes**

CBCLi = CBCL internalizing score. CBCLe = CBCL externalizing score. Associations between sleep variables of interest and behavioral reports on CBCL within groups. Association statistics (F-value), *p*-values, and significance controlling for False Discovery Rate (FDR) with the Benjamini-Hochberg Procedure are presented for unadjusted model and adjusted model with standard NVIQ and any medication use. Bolded *p*-values represent significance *p* < 0.05.

## Data Availability

The data are not publicly available as informed consent/assent was not obtained from participants for data sharing.
